# Cardiac Computed Tomography Radiomics-Based Approach for the Detection of Left Ventricular Remodeling in Patients with Arterial Hypertension

**DOI:** 10.3390/diagnostics12020322

**Published:** 2022-01-27

**Authors:** Armando Ugo Cavallo, Jacopo Troisi, Emanuele Muscogiuri, Pierpaolo Cavallo, Sanjay Rajagopalan, Rodolfo Citro, Eduardo Bossone, Niall McVeigh, Valerio Forte, Carlo Di Donna, Francesco Giannini, Roberto Floris, Francesco Garaci, Massimiliano Sperandio

**Affiliations:** 1 Department of Biomedicine and Prevention, University of Rome “Tor Vergata”, 00133 Rome, Italy; carlo.didonna@students.uniroma2.eu (C.D.D.); roberto.floris@uniroma2.it (R.F.); francesco.garaci@uniroma2.it (F.G.); 2 Division of Radiology, San Carlo di Nancy Hospital, GVM Care and Research, 00165 Rome, Italy; valerio.forte@gmail.com (V.F.); msperandio@gvmnet.it (M.S.); 3 Department of Medicine, Surgery and Dentistry, “Scuola Medica Salernitana”, University of Salerno, 84100 Salerno, Italy; jtroisi@unisa.it or; 4 Theoreo srl—Spin-Off Company of the University of Salerno, 84100 Salerno, Italy; 5 Radiology Department, Ospedale S. Andrea, Sapienza—Università di Roma, 00189 Rome, Italy; e.muscogiuri@gmail.com; 6 Department of Physics “E.R. Caianello”, University of Salerno, 84100 Salerno, Italy; pcavallo@unisa.it; 7 Istituto Sistemi Complessi—Consiglio Nazionale delle Ricerche (CNR), 00185 Rome, Italy; 8 Division of Cardiovascular Medicine, Harrington Heart and Vascular Institute, Cleveland, OH 44106, USA; sxr647@case.edu; 9 Division of Cardiology, University Hosptal “San Giovanni di Dio e Ruggi D’Aragona”, 84100 Salerno, Italy; rodolfo.citro@sangiovannieruggi.it; 10 Cardiology Division, “A. Cardarelli” Hospital, 80131 Naples, Italy; eduardo.bossone@aocardarelli.it; 11 Department of Radiology, St Vincent’s University Hospital, Merrion Road, D04 T6F4 Dublin, Ireland; mcveighn@tcd.ie; 12 School of Medicine, University College Dublin, D04 T6F4 Dublin, Ireland; 13 Division of Cardiology, Maria Cecilia Hospital, GVM Care and Research, 48033 Cotignola, Italy; fgiannini@gvmnet.it; 14 San Raffaele Cassino, 03043 Cassino, Italy

**Keywords:** Cardiac Computed Tomography, hypertension, radiomics, machine learning, remodeling

## Abstract

The aim of the study is to verify the feasibility of a radiomics based approach for the detection of LV remodeling in patients with arterial hypertension. Cardiac Computed Tomography (CCT) and clinical data of patients with and without history of arterial hypertension were collected. In one image per patient, on a 4-chamber view, left ventricle (LV) was segmented using a polygonal region of interest by two radiologists in consensus. A total of 377 radiomics features per region of interest were extracted. After dataset splitting (70:30 ratio), eleven classification models were tested for the discrimination of patients with and without arterial hypertension based on radiomics data. An Ensemble Machine Learning (EML) score was calculated from models with an accuracy >60%. Boruta algorithm was used to extract radiomic features discriminating between patients with and without history of hypertension. Pearson correlation coefficient was used to assess correlation between EML score and septum width in patients included in the test set. EML showed an accuracy, sensitivity and specificity of 0.7. Correlation between EML score and LV septum width was 0.53 (*p*-value < 0.0001). We considered LV septum width as a surrogate of myocardial remodeling in our population, and this is the reason why we can consider the EML score as a possible tool to evaluate myocardial remodeling. A CCT-based radiomic approach for the identification of LV remodeling is possible in patients with past medical history of arterial hypertension.

## 1. Introduction

Radiomics is defined as the quantitative extraction and analysis of data from medical images, that are translated into a high-dimensional mineable feature space using dedicated automatized feature extraction algorithm [1]. This process underlies the concept that advanced quantitative texture analysis (TA) of biomedical images can provide additional information that is undetectable by the human eye but are already within the digital data of composed medical images [2]. Furthermore, radiomics allows the extraction of a large number of features from a digital image to find characteristic features that can be useful for diagnosis and prognosis of a wide variety of diseases.

Cardiac Computed Tomography (CCT) has proven to be an optimal modality on which to perform TA. It has a rapid acquisition time with images of suitable quality resolution and has frequent use in clinical practice resulting in large datasets of readily available CCT images [3]. Radiomics has already proven to improve CCT performance for detection of advanced coronary atherosclerotic lesion, characterization of atherosclerotic plaques, evaluation of coronary stenosis and characterization of the myocardium [4,5,6,7].

The most common clinical use of CCT is the assessment of coronary artery disease (CAD). This is a multi-factorial disease with many patients having multiple co-morbidities such as hypertension, hypercholesterolemia or metabolic syndrome.

Hypertension is the leading risk factor for cardiovascular complications and commonly is associated with adverse structural heart remodeling, leading LV disfunction and hypertensive cardiomyopathy [8]. Advanced quantitative TA of the myocardium may provide additional information that cannot be assessed by visual analysis such as fibrosis, myocyte hypertrophy and scar tissue which may have major prognostic implications [2].

The aim of our research was to test the feasibility of extracting CCT-derived radiomics features for the detection of LV remodeling in patients with arterial hypertension.

## 2. Materials and Methods

### 2.1. Population and Study Design

In this retrospective study, images of patients undergoing CCT between January to September 2020 at San Carlo di Nancy Hospital (Rome, Italy) were analyzed. For the purpose of this study, images of patients with and without prior diagnosis of arterial hypertension meeting the inclusion criteria were considered. Arterial hypertension was defined as per the European Society of Hypertension guidelines [9]. The inclusion criteria included: age >18 years, absence of coronary stenosis >50%, absence of clinical or imaging criteria for other types of cardiomyopathies (e.g., dilated, hypertrophic), absence of respiratory or heart rate related artifacts on CT images.

### 2.2. Data Collection

Demographic and clinical characteristics (see Table 1) were collected at enrollment for each patient included in the study and recorded on a dedicated database. Subjects re-porting a past smoking history were considered as smokers. Co-morbidities including diabetic patients and of dyslipidemia status was also collected. Prior to imaging both systolic and diastolic pressure were measured and recorded according to European Society of Hypertension guidelines.

### 2.3. Image Acquisition and Analysis

CCT images were obtained using a 512-slice scanner (Revolution CT, General Electric, Chicago, IL, USA), with prospective gating, setting the slice thickness at 0.625 mm, 120 kV, automatic mA, rotation time of 0.28 s, DFOV: 25 cm, Detector Coverage: 160 mm. All patients were subjected to administration of contrast material (Omnipaque 350 mg/mL, GE Healthcare, Chicago, IL, USA) at a rate of 5 mL/s followed by a saline chaser. The image acquisition was triggered after a threshold of 80 HU was reached in a region of interest placed in the left ventricle (bolus-tracking technique).

**Figure 1:**
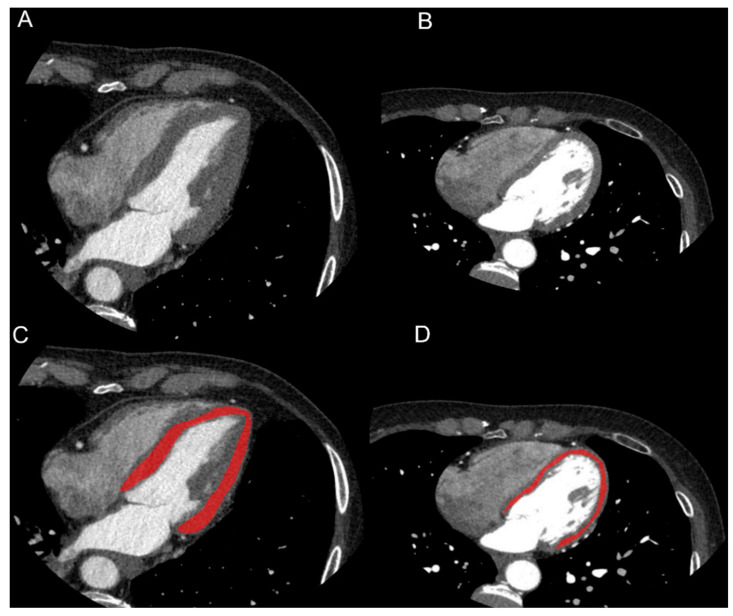
Images and segmentation examples of cardiac CTs of patient with history of hypertension (HTN, (**A**–**C**)) and without history of hypertension (NC, (**B**–**D**)). On a 4-chamber view, LV was segmented using a polygonal region of interest (ROI). Care was taken in not including blood in LV cavity, epicardial fat or major coronary arteries. Clinical data of HTN patient were: male, 73 years old, BMI = 24.7 kg/m^2^, history of diabetes, dyslipidemia and hypertension, septum width: 14 mm. Clinical data of NC patient were: female, 61 years old, BMI = 18.9 kg/m^2^, smoker, familiarity with cardiovascular disease, no history of diabetes, dyslipidemia and hypertension, septum width: 8 mm.

In one image per patient, on a 4-chamber view and at 75% of R-R interval, LV was segmented using a polygonal region of interest (ROI) by two radiologists in consensus. Care was taken to not include blood in LV cavity, epicardial fat, myocardial trabeculations or major coronary arteries in the ROI (Figure 1).

Gray-level normalization was performed by rescaling the histogram data within μ-gray-level mean ±3 σ (σ-gray-level standard deviation) to reduce contrast and brightness variations that might impair texture feature quantification [10].

A total of 377 features per ROI were extracted using MaZDa ver. 4.6 software (Institute of Electronics, Technical University of Lodz, Lodz, Poland) [11]. Texture features were calculated from geometry, the gray-level histogram analysis, co-occurrence matrix calculated in five distances, run-length matrix calculated in four directions, auto-regressive model, and Wavelet transform. The detailed description of the radiomic features is freely available on the online MaZda 4.6 manual.

### 2.4. Statistical Analysis

Clinical data distribution was analyzed using the D’Agostino–Pearson test. Student’s *t*-test was used to determine the *p*-value assessing the significance of the difference between the investigated classes (α-value < 0.05). *p*-value was corrected according to the false discovery rate (FDR) using the Benjamini–Hochberg procedure. Categorical variables were compared using the χ^2^-test. Radiomics data were scaled by an autoscaling process (mean-centered and divided by standard deviation of each variable). Pearson correlation coefficient was used to assess correlation between continuous variables.

#### 2.4.1. Classification Models

The auto-scaled dataset containing the texture analysis results, was first divided in two parts (70:30 ratio), one used to train the classification models and one to test them and to evaluate the model performances. In each dataset, the classes were quite evenly represented. Eleven classification models were trained and included: Partial Least Square Discriminant Analysis (PLS-DA), Naïve Bayes (NB), Generalized Linear Model (GLM), Logistic Regression (LR), Fast Large Margin (FML), Deep Learning (DL), Decision Tree (DT), Random Forest (RF), Gradient Boosted Trees (GBT), artificial Neural Network (aNN) and Support Vector Machine (SVM). Hyperparameter tuning was also applied searching the setting able to maximize each model’s accuracy. Overfittings were evaluated using a cross-validation procedure based on training dataset. Furthermore, models were trained using many features subsets. Features were first screened using 3 criteria: (a) correlation (features that too closely, or not at all, mirror the image labels) (correlation < 0.001% or >95%, (b) stability (features with almost all identical values) (>90% identical values), (c) missing (features with missing values). Features that did not fulfil the screening criteria were deemed unsuitable and removed from the dataset to avoid overfitting and maximize the model’s accuracy.

#### 2.4.2. Ensemble Machine Learning Score (EML Score)

Receiver operating characteristic (ROC) curves, sensitivity, specificity, positive and negative predictive values, positive and negative likelihood ratios, and accuracy were measured to assess the ability of the EML score to correctly classify the images. A cut-off point was evaluated as the score maximizing the Youden’s Index (sensitivity + specificity − 1). DeLong non-parametric approach was used to compare the area under the curve receiver operating characteristic (AUCROC).

Models showing an accuracy higher of 60% were selected and ensembled using a voting scheme assuming both cross-validation accuracy and confidence (i.e., distance from classification margin) as a vote weight. Ensembling was performed according to Cavallo et al. [12]. Briefly, for the images classified as derived from subjects with Hypertension (HTN), the scores (the products of model accuracy and classification confidences) were left as is, while for each Controls (NC) classification the scores were multiplied by −1. Finally, an Ensemble Machine Learning (EML) score was calculated for each sample by summing all the single model classification scores. Additional information on EML score is available in Supplementary Materials File S1.

The overall diagnostic performance of the proposed score was investigated using a confusion matrix to summarize the results obtained using samples in the test set.

Statistical analyses were performed using Rapid Miner Studio v9.8.001 (RapidMiner GmbH, Boston, MA, USA), and R v4.0.4 (R. R Development CORE TEAM, R: A language and environment for statistical computing, R Foundation for Statistical Computing Vienna, Austria, 2008).

### 2.5. Relevant Features Selection

#### 2.5.1. Smile Plot

Radiomic features were investigated by means of smile plot obtained by plotting for each feature the negative log of the p-value on the *y* axis and the log of the fold change (FC) on the *x* axis. This resulted in data points with low *p*-values (highly significant) appearing towards the higher end of the plot and data points with higher FC at the plot margins. The log of the FC was used so that any change appeared equidistant from the center in both directions. By plotting the data in such a manner two regions of interest in the plot were delineated: at the higher end of the plot and at the left- or right margins of the plot. Any point in both locations on the plot represented values that displayed large magnitude FCs (located at the left or right plot margins) as well as high statistical significance (hence being toward the higher end of the plot).

#### 2.5.2. Boruta Algorithm (BA)

Boruta algorithm was also used to evaluate the relevance of each radiomics feature. From the radiomics’ dataset (DSet) a new dataset (shadow dataset ShDSet) was created randomly shuffling each feature. ShDSet was attached to DSet to obtain a new dataset (Boruta dataset BaDSet), which has twice the number of columns of DSet.

A Random Forest algorithm was trained using BaDSet, and the information gain was evaluated for all features. A threshold level was defined as the higher information gain among the features derived from ShDSet. All the features with a lower information gain were excluded. The whole process was repeated 2000 times.

## 3. Results

CCT images from 158 patients, 83 with history of arterial hypertension (mean age 65.63 ± 10.23 years, Females = 42.17%) and 75 without (mean age = 55.59 ± 12.42 years, Females = 60%) were used to perform image analysis.

Age, BMI, history of diabetes and LV septum thickness were higher in HTN patients, as was male sex (Table 1). The difference between other clinical investigated variables were not statistically significant.

**Figure 2:**
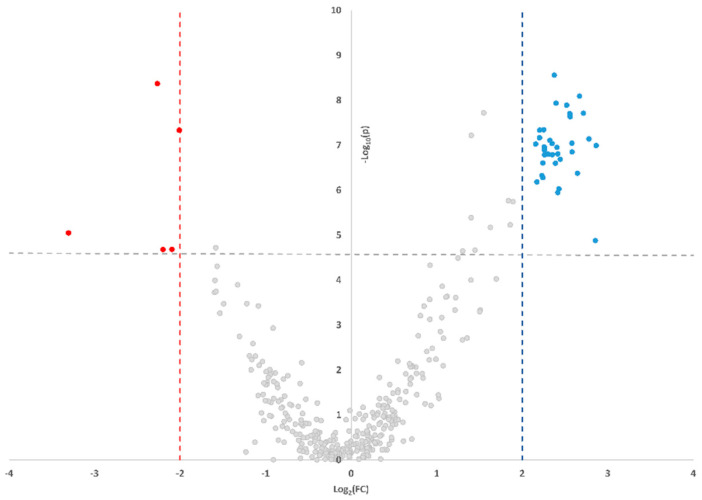
Smile plot reporting the features’ values change between HTN and NC patients. Red dots indicate features reduced in HTN, while blue dots indicate features increased in HTN.

Exploratory analysis of the 377 radiomic features showed that 43 (11.4%), showed a fold change higher of ±2 and an FDR corrected p-value in patients with HTN (Figure 2). There were 23 features selected with the Boruta algorithm (Table 2).

Classification performances evaluated on test set are reported in Table 3. Naïve Bayes, Logistic Regression, Deep Learning, Decision Tree, artificial Neural Network and Support Vector Machine resulted in an accuracy less than 60% (data not shown) and were not further used to train the EML.

**Figure 3:**
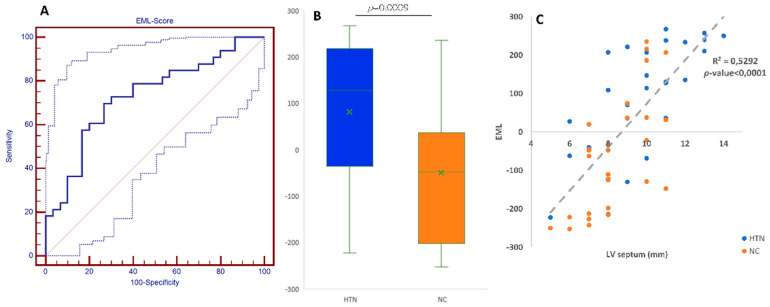
(**A**) Area under the receiver operating characteristic curve of the EML score (0.731 ± 0.064) (*p*-value < 0.001; (**B**) EML-score distribution among the HTN and NC patients; means values resulted 82.0 ± 155.9 and −49.1 ± 152.7, respectively (*p*-value < 0.001); (**C**) Correlation among LV septum width (mm) and Ensemble Machine Learning Score (R^2^ = 0.5292, *p*-value < 0.0001).

Accuracy of the selected models (GLM, FLM, RF, GBT and PLS-DA) ranged from 66.4% to 80.0%. Ensemble models showed 70.0% accuracy. Sensitivity and specificity resulted balanced for all models, ranging between 54–87% and 45–77%, respectively (Table 3). Youden’s evaluations of the best cut-off were performed for the EML resulting in 31.98. The area under the receiver operating characteristic curve (AUCROC) resulted in 0.731 ± 0.064 for the EML score (*p*-value < 0.001) (Figure 3A). Moreover, Figure 3B shows the EML score distribution among the HTN and NC patients which means that values resulted 82.0 ± 155.9 and −49.1 ± 152.7, respectively (*p*-value < 0.001). Moreover, the EML score was well correlated with the LV septum width (R^2^ = 0.5292, *p*-value < 0.0001) (Figure 3C).

**Figure 4:**
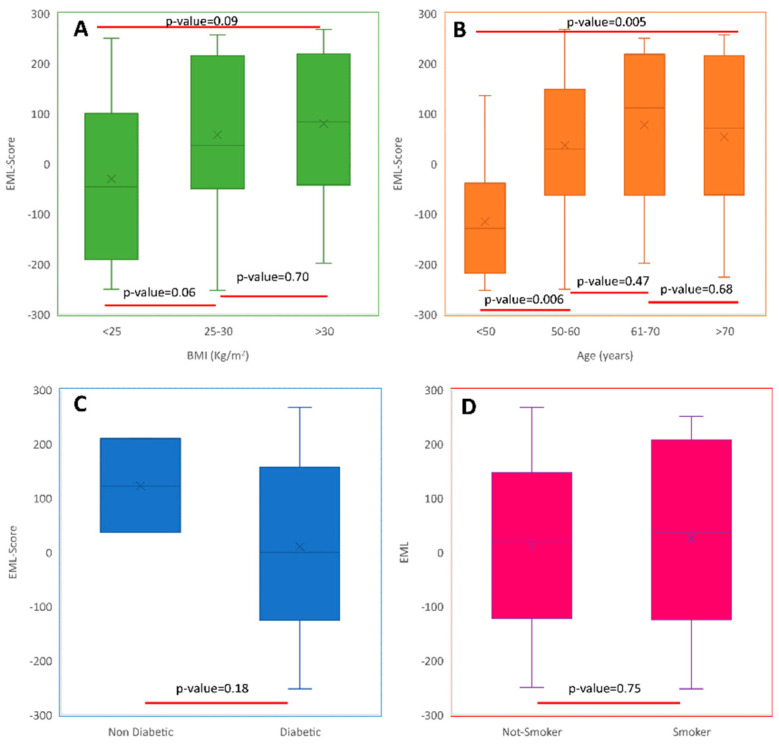
Box and whiskers plot reporting the EML score distribution in (**A**) normal weight (BMI < 25 kg/m^2^), overweight (25 < BMI < 30) and obese (BMI > 30 kg/m^2^) subjects; (**B**) subjects with <50 years, 51–60, 61–70 and >70 years; (**C**) in diabetic and non-diabetic subjects; (**D**) in smoker and not-smoker subjects.

The EML score was also evaluated in diabetic and non-diabetic enrolled subjects as well as in the subjects with less of 50, 50–60, 61–70 and more than 70 years. The EML score was lower in the youngest cohort (<50 years) only (*p* = 0.006). Moreover, BMI did not affect the EML score, with no difference (*p* > 0.05) among normal weight (BMI < 25 kg/m^2^), overweight (25 < BMI < 30) and obese (BMI > 30 kg/m^2^) subjects (Figure 4).

## 4. Discussion

Our results suggest multiple alterations in the radiomic features in the myocardium of patients with history of HTN undergoing a CCT for routine coronary angiography. These findings might suggest structural alterations in the myocardium of these patients. In our analysis, multiple Entropy and Gray Level non-Uniformity (RLN) feature parameters, which are a measure of intrinsic randomness/variability in neighborhood intensity value differences were selected by the Boruta algorithm. The Boruta algorithm ruled out four different RLN features (Horizontal, Vertical, 135 degree and 45 degree), that also showed a statistically significant difference between groups. RLN is a measure of the similarity of run lengths throughout the image. The lower the RLN value, the more homogeneity among run lengths in the image is present. Patients with arterial hypertension showed higher RLN values compared to patients without arterial HTN (Table 2), indicating a more homogeneous distribution of gray levels among LV pixels. This may reflect a higher myocardial architectural distortion in the LV of patients with arterial hypertension, which is consistent with our hypothesis. There was also a significant difference in the complex geometry features of the LV, reflecting a different LV shape associated to hypertensive remodeling.

Our results encourage an approach where a set of distinct radiomic features may be used to train an ensemble machine learning model with an overall accuracy of 70%, and relatively good sensitivity, specificity, positive and negative predictive values.

The practical application of such an approach may lie in its ability to discriminate patients with LV remodeling from hypertension even without a past medical history of arterial hypertension using CT instead of cardiac MRI (CMR), which is currently the method of choice for assessing myocardial fibrosis through measures such as myocardial extracellular volume fraction (ECV) which quantifies the interstitial space. The latter is, however, an indirect measure that relies on the extravasation of gadolinium, a pure extravascular contrast agent, into the interstitial space and measurements of T1 relaxation before and after contrast administration. In the absence of myocardial edema or amyloidosis, ECV is an adequate measurement of diffuse myocardial fibrosis [13]. CCT could be useful in the near future to provide additional prognostic information by detecting myocardial fibrosis in several cardiovascular pathologies that are already primarily diagnosed with CCT imaging such as CAD, cardiomyopathies, coronary artery anatomy and valvopathies.

Recent advances suggest that LV fibrosis is the end-stage of a number of myocardial processes including obesity, diabetes mellitus, sex, age, environmental exposures and genetic factors which collectively, through pathways of inflammatory stress, may eventually lead to fibrosis and LV dysfunction and hypertensive cardiomyopathy [14,15]. Indeed, fibrosis is thought to precede left ventricular hypertrophy. This theory may explain why many patients with high blood pressures do not present with clinically detectable LV hypertrophy. The process of fibrosis in recent years has been shown to be powerfully driven by the interplay of cardiomyocytes, resident cells of the myocardium (i.e., fibroblasts, endothelial cells, pericytes) and immune cells. The latter may be recruited from the circulation (e.g., immune and inflammatory cells and progenitor cells) in response to a variety of stimuli [16].

Our analysis showed a significant correlation between EML score and septum width (R2 = 0.53, *p*-value < 0.0001), independent of diabetes, BMI and age in those above the age of 50 years old. The significantly low EML score in the diabetic vs. non-diabetic cohort suggests that either cardiac remodeling by arterial hypertension is a long-term disease process or that the effect of diabetes on the myocardium is more significant in younger patients. This finding adds additional information on the potential role of machine learning and texture analysis in detecting myocardial modifications in CCT images.

This study has several important limitations that must be acknowledged: (1) the absence of a reference standard (CMR) for the quantification of LV remodeling and fibrosis; (2) retrospective nature of analysis; (3) patients with hypertension were on antihypertensive medication for a variable period of time (ranging from 1 to >20 years) which may affect cardiac remodeling; (4) the possibility of systematic differences related to the size of segmented volumes must be considered, especially for higher order features; (5) blood pressure control, type of antihypertensive medications, as well as control of comorbidities, were not assessed; (6) Clinical data of HTN and NC patients showed some statistically significant difference that might affect outcomes; for the EML we’ve taken into account only radiomics data in order to minimize possible biases.

In summary, our study has identified a possible radiomic-based approach for the identification of LV remodeling in patients with arterial hypertension and potential radiomic features associated with LV remodeling in patients with this disease.

We considered LV septum width as a surrogate of myocardial remodeling in our population, and this is the reason why we can consider the EML score as a possible tool to evaluate myocardial remodeling.

Further confirmation of our findings will be needed also for other conditions associated with LV fibrosis, and in comparison with imaging modalities such as echocardiography and cardiac MRI.

## Figures and Tables

**Table 1:** Baseline clinical features of selected patients. HTN: hypertension; NC: controls.

	HTN (*n* = 83)	NC (*n* = 75)	*p*-Value
Sex (F) (%)	35 (42.17%)	45 (60%)	0.038
Age	65.63 ± 10.23	55.59 ± 12.42	<0.001
Dyslipidemia	18 (21.7%)	10 (13.3%)	0.24
BMI (kg/m^2^)	28.4 ± 6.1	25.5 ± 4.9	<0.001
Diabetes (%)	18 (21.7%)	4 (5.12%)	0.006
LV Septum Width (mm)	10.01 ± 2.7	8.15 ± 1.66	<0.001
Systolic blood pressure (mmHg)	131.6 ± 14.2	124 ± 11.8	0.06
Diastolic blood pressure (mmHg)	77.6 ± 8.18	75.8 ± 10.1	0.3

**Table 2:** Radiomics’ features selected with Boruta algorithm. HTN: patients with history of hypertension; NC: controls; Geo: Geometry features; DifEntrp: Difference Entropy; RLNonUni: Run Length Non-Uniformity; GrNonZeros: Percentage of Pixels with Nonzero Gradient; WavEnLH: Wavelet Energy.

	HTN	NC	*p*-Value
GeoF	3495 ± 1246.18	2514.85 ± 735.74	<0.001
GeoSxL	18,978.13 ± 4315.37	18,074.65 ± 8676.49	0.005
GeoW3	1143.16 ± 329.01	1362.56 ± 357.21	<0.001
GeoW5b	0.023 ± 0.01	0.019 ± 0.005	<0.001
GeoW12	0.47 ± 0.16	0.37 ± 0.12	<0.001
GeoEl	2.17 ± 0.82	1.55 ± 0.48	<0.001
S(1,0)DifEntrp	1.2 ± 0.04	1.19 ± 0.05	0.6
S(1,−1)DifEntrp	1.31 ± 0.04	1.3 ± 0.04	0.7
S(2,0)DifEntrp	1.4 ± 0.04	1.38 ± 0.05	0.07
S(0,2)DifEntrp	1.39 ± 0.04	1.38 ± 0.05	0.77
S(2,2)DifEntrp	1.44 ± 0.04	1.43 ± 0.05	0.2
S(2,−2)DifEntrp	1.44 ± 0.04	1.43 ± 0.04	0.3
S(3,0)DifEntrp	1.45 ± 0.04	1.44 ± 0.04	0.2
S(3,3)DifEntrp	1.45 ± 0.04	1.44 ± 0.04	0.1
S(4,0)DifEntrp	1.46 ± 0.04	1.45 ± 0.04	0.5
S(5,0)DifEntrp	1.45 ± 0.03	1.45 ± 0.04	0.6
S(5,−5)DifEntrp	1.46 ± 0.03	1.44 ± 0.04	0.04
Horzl_RLNonUni	2939.47 ± 1051.8	2090.89 ± 646.64	<0.001
Vertl_RLNonUni	2904.91 ± 1033.5	2078.15 ± 637.73	<0.001
45dgr_RLNonUni	3045.01 ± 1080.71	2171.12 ± 664.71	<0.001
135dr_RLNonUni	3083.88 ± 1106.38	2202.61 ± 665.95	<0.001
GrNonZeros	0.98 ± 0.01	0.98 ± 0.01	0.8
WavEnLH_s-4	424.46 ± 111.54	465.10 ± 116.70	0.027

**Table 3:** Diagnostic performance of each classification model and of the ensemble. Results are expressed as value ± standard error.

	S	Sp	PPV	NPV	PLR	NLR	Accuracy
GLM	0.70 ± 0.10	0.64 ± 0.10	1.91	0.48	0.67 ± 0.10	0.67 ± 0.10	0.667
FLM	0.54 ± 0.10	0.76 ± 0.09	2.28	0.60	0.72 ± 0.11	0.59 ± 0.0.9	0.664
RF	0.87 ± 0.07	0.45 ± 0.11	1.59	0.29	0.63 ± 0.09	0.77 ± 0.12	0.667
GBT	0.78 ± 0.09	0.55 ± 0.11	1.72	0.40	0.64 ± 0.09	0.71 ± 0.11	0.667
PLS-DA	0.83 ± 0.08	0.77 ± 0.09	3.63	0.23	0.79 ± 0.08	0.81 ± 0.09	0.800
Ensemble	0.70 ± 0.08	0.70 ± 0.08	2.32	0.43	0.72 ± 0.08	0.68 ± 0.08	0.7

Abbreviation: S = Sensitivity; Sp: Specificity; PPV: Positive Prognostic Value; NPV: Negative Prognostic Value; PLR: Positive Likelihood Ratio; NLR: Negative Likelihood Ratio; GLM: Generalized Linear Model; FLM: Fast Large Margin; RF: Random Forest; GBT: Gradient Boosted Trees; PLS-DA: Partial Least Square Discriminant Analysis.

## Data Availability

The data sets generated and analyzed during the current study are available from the corresponding author on reasonable request.

## Supplementary Materials

The following are available online at https://www.mdpi.com/article/10.3390/diagnostics12020322/s1, File S1: Title: Ensemble Machine Learning Score.
