# Supplementary material for: Cardiac Computed Tomography Radiomics-Based Approach for the Detection of Left Ventricular Remodeling in Patients with Arterial Hypertension
Source: Diagnostics (Basel). 2022 Jan 27;12(2):322. doi: 10.3390/diagnostics12020322 (PMC8871253; doi:10.3390/diagnostics12020322)
Supplement: Supplementary file 1 [file diagnostics-12-00322-s001.zip › diagnostics-1539374-supplementary.pdf]

Ensemble Machine Learning Score

In machine learning (ML), classification refers to the process of predicting the class of given data points.

Classifiers may produce different predictions with different ability to predict class in different datasets.

Ensembling is performed to increase and uniform accuracy, sensitivity and specificity of different classification ML models and consists in using predictions from different classifiers to obtain a stronger and more robust result.

In our study, after images classification and radiomics features extraction, we trained different ML models and selected ones with accuracy on test set >60%.

For the Ensembling Machine Learning (EML) score generation, each model accuracy was multiplied by the classification confidence and, for the images classified as derived from subjects with Hypertension, the scores were left as is, while for each normal subject scores were multiplied by -1.

Finally, the (EML)-score was calculated by summing all the single model classification scores.

Receiver operating characteristic (ROC) curves, sensitivity, specificity, positive and negative predictive values, positive and negative likelihood ratios, and accuracy were measured to assess the ability of the EML-score to correctly classify the images.

A cut-off point was evaluated as the score maximizing the Youden's Index (sensitivity +specificity -1).

Youden's evaluations of the best cut off were performed for the EML resulting in 31.98. Area under the receiver operating characteristic curve (AUCROC) resulted 0.731±0.064 for the EML-score (p-value<0.001) with an accuracy of 70% on the test set.

For more information about EML score and its application please refer to:

1. Dietterich TG (2000) Ensemble Methods in Machine Learning. Lect Notes Comput Sci (including Subser Lect Notes Artif Intell Lect Notes Bioinformatics) 1857 LNCS:1–15. https://doi.org/10.1007/3-540-45014-9_1

2. Cavallo AU, Troisi J, Forcina M, et al (2021) Texture Analysis in the Evaluation of Covid-19 Pneumonia in Chest X-Ray Images: a Proof of Concept Study. Curr Med Imaging Former Curr Med Imaging Rev 17:. https://doi.org/10.2174/1573405617999210112195450

3. Troisi J, Raffone A, Travaglino A, et al (2020) Development and Validation of a Serum Metabolomic Signature for Endometrial Cancer Screening in Postmenopausal Women. JAMA Netw open 3:e2018327. https://doi.org/10.1001/jamanetworkopen.2020.18327
